# A Syntactic Information–Based Classification Model for Medical Literature: Algorithm Development and Validation Study

**DOI:** 10.2196/37817

**Published:** 2022-08-02

**Authors:** Wentai Tang, Jian Wang, Hongfei Lin, Di Zhao, Bo Xu, Yijia Zhang, Zhihao Yang

**Affiliations:** 1 College of Computer Science and Technology Dalian University of Technology Dalian China

**Keywords:** medical relation extraction, syntactic features, pruning method, neural networks, medical literature, medical text, extraction, syntactic, classification, interaction, text, literature, semantic

## Abstract

**Background:**

The ever-increasing volume of medical literature necessitates the classification of medical literature. Medical relation extraction is a typical method of classifying a large volume of medical literature. With the development of arithmetic power, medical relation extraction models have evolved from rule-based models to neural network models. The single neural network model discards the shallow syntactic information while discarding the traditional rules. Therefore, we propose a syntactic information–based classification model that complements and equalizes syntactic information to enhance the model.

**Objective:**

We aim to complete a syntactic information–based relation extraction model for more efficient medical literature classification.

**Methods:**

We devised 2 methods for enhancing syntactic information in the model. First, we introduced shallow syntactic information into the convolutional neural network to enhance nonlocal syntactic interactions. Second, we devise a cross-domain pruning method to equalize local and nonlocal syntactic interactions.

**Results:**

We experimented with 3 data sets related to the classification of medical literature. The F1 values were 65.5% and 91.5% on the BioCreative ViCPR (CPR) and Phenotype-Gene Relationship data sets, respectively, and the accuracy was 88.7% on the PubMed data set. Our model outperforms the current state-of-the-art baseline model in the experiments.

**Conclusions:**

Our model based on syntactic information effectively enhances medical relation extraction. Furthermore, the results of the experiments show that shallow syntactic information helps obtain nonlocal interaction in sentences and effectively reinforces syntactic features. It also provides new ideas for future research directions.

## Introduction

**Figure 1:**
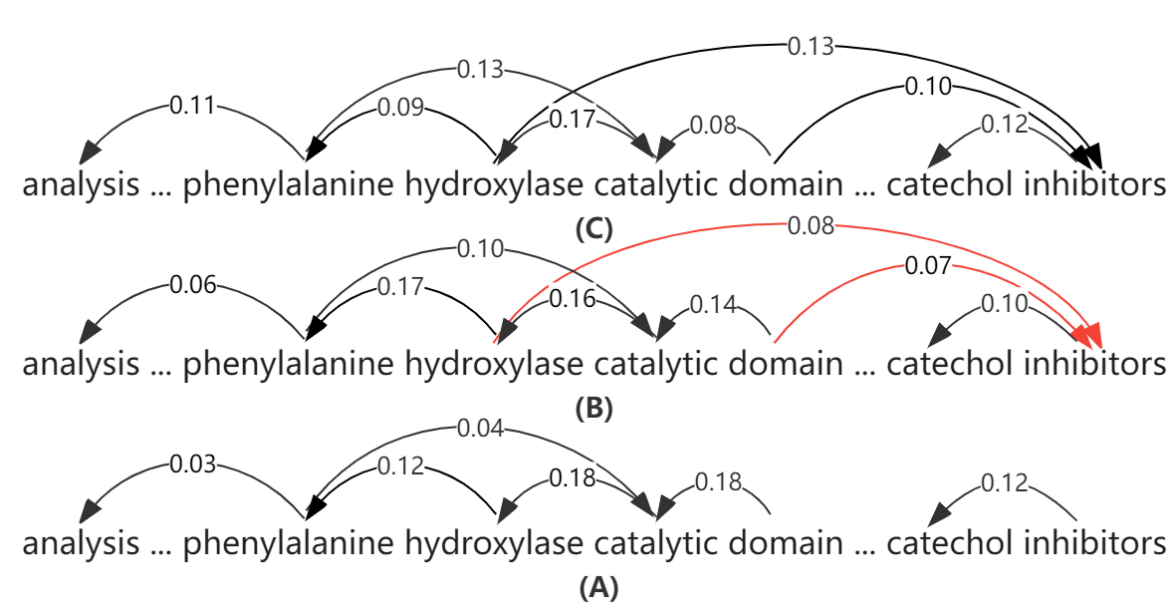
Interaction features by introducing shallow syntactic information and equalization. (A) Dependency tree without processing; (B) dependency tree after syntactic structure fusion; and (C) dependency tree after the pruning process. The weight of each arc in the forest is indicated by its number. Some edges were omitted for the sake of clarity.

The classification of medical literature is especially necessary in light of the ever-increasing volume of material. Medical relation extraction is a typical method for classifying medical literature, which classifies the literature quickly by using medical texts. The advancement of this technology will have a profound impact on medical research. For example, in the sentence, “The catalytic structural domain of human phenylalanine hydroxylase binds to a catechol inhibitor,” from the medical literature (Figure 1), there is a “down-regulated” relation (CPR:4). We can input the text into the model to obtain the relation category as “CPR:4” in the CPR data set. Thus, we can quickly classify medical literature.

There are 2 primary approaches for extracting medical relations: network-based and rule-based approaches. Rule-based models only obtain shallow syntactic information by imposing rule constraints, leading to early studies that focus on obtaining shallow syntactic information, such as part-of-speech tags [1] or a complete structure [2]. In contrast, the neural network–based model focuses on syntactic dependency features but leaves out shallow syntactic information. Now, large-scale neural network models have significantly outperformed rule-based models with the resurgence of neural network approaches [3]. As a result, researchers no longer value shallow syntactic information, and medical relation extraction is gradually adopting a neural network approach. Early efforts leverage graph long short-term memory (LSTM) [4] or graph neural networks [5] to encode the 1-best dependency tree in the medical relation extraction. Zhang et al [6] analyzed sentence interaction information using a graph convolutional network (GCN) model [7]. Song et al [8] constructed a dependency forest, and Jin et al [9] concurrently trained a relation extraction model and a pretrained dependency parser [10] to mitigate error propagation when incorporating the dependency structure.

In medical relation extraction, both rule-based and neural network–based models have drawbacks. First, the rule-based approach is too costly to design rules for medical texts. Because the customization of medical text rules is different from the general-purpose domain [11], it relies more on expert knowledge. Second, the neural network–based approach has difficulty in capturing sufficient syntactic features [12], as shallow syntactic information is discarded. As a result, we designed a soft-rule neural network model that allows the encoding phase of the neural network model to carry shallow syntactic features, overcoming the problem of insufficient syntactic features after the neural network discards the rules.

Our model can better capture the interaction features in sentences by introducing shallow syntactic information and equalization. As we can see, Figure 1 shows the unprocessed sentence (Figure 1A). With the addition of shallow syntactic information to the model, it becomes the sentence shown in Figure 1B with the addition of hydroxylase and inhibitor interactions. When the model is equalized, Figure 1B transforms into Figure 1C, with a more evenly distributed score of weight interactions within sentences.

Overall, we propose a syntactic feature–based relation extraction model for medical literature classification, where shallow syntactic information is incorporated and equalized in a neural network. First, our model's encoder is the ordered neuron LSTM (ON-LSTM) [13]. When encoded, it captures the syntactic structure in the shallow syntactic information [13]. Second, we design a pruning process on the attention matrix to balance the weight of sentence interactions.

## Methods

### Settings

#### Overview

We chose 3 data sets from the medical field to evaluate our model. Using the data sets, we experimented with 2 types of medical relation extraction tasks at the cross-sentence and sentence levels.

#### Extraction of Cross-sentence Relations

For extracting cross-sentence relations, 6086 binary relation instances were extracted from PubMed [4] and 6986 ternary relation instances were noted in the data sets. This yielded 2 data sets for more detailed evaluation [14]: one contains 5 categories of relational labels and the other groups all labels that are not “None” into one category.

For extracting sentence-level relation. We referred to the BioCreative ViCPR (CPR) and Phenotype-Gene Relationship (PGR) data sets. The PGR data set introduces the information between human genes with human phenotypes; it contains 218 test instances and 11,781 training instances and 2 types of relation labels: “No” and “Yes.” The CPR data set contains information about the interactions between human proteins and chemical components. It has 16,106 training, 14,268 testing, and 10,031 development instances, as well as containing 5 relations such as “None,” “CPR:2,” and “CPR:6” relation. We combined these 2 data sets into 1 table to make it more intuitive.

#### Experimental Parameter Setting

For the cross-sentence relation task, we referred to the same data divides that Guo et al [14] used. The hidden size of ON-LSTM is set to 300 in our stochastic gradient descent optimizer with a 300-dimensional Glove and 0.9 decay rate and reports the average test accuracy over 5 cross-validation folds. For the sentence-level task, the F1 results are shown [8], and we randomly divided 10% of the PGR training set as the development set to ensure consistent data division. We fine-tuned the hyperparameters based on the outcomes of the development sets. The results marked with an asterisk are based on a reimplementation of the original model. The aforementioned configuration ensures that our model has a consistent data partitioning and operating environment with the baseline.

### The Overall Architecture

**Figure 2:**
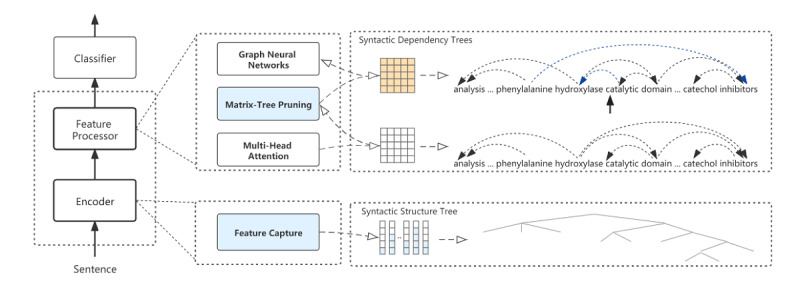
Diagrammatic representation of the syntactic enhancement graph convolutional network model showing an instance and its syntactic information processing flow. The syntactic structure tree can be obtained from the encoder, and a matrix-tree can transform the syntactic dependency tree in the feature processor.

An overview of our proposed syntactic enhancement graph convolutional network (SEGCN) model (Figure 2) consists of 3 parts: an Encoder, a Feature Processor, and a classifier. The Encoder incorporates the syntactic structural features, and the Feature Processor handles the features containing structural information.

### Encoder

We used ON-LSTM [13] to obtain a syntactic structure in shallow syntactic information. The ON-LSTM introduces syntactic structure information while encoding by layering the neurons. In terms of the overall framework, it is similar to LSTM. Here, we mathematically illustrate how ON-LSTM incorporates syntactic structural features.

Given a sentence *s* = *x*_1_,…,*x*_n_, where *x*_i_ represents the *i*-th word. We have written **h** = **h**_1_,…,**h**_n_ for the structural output of the sentence **h** 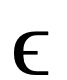 R^n×^*^d^*, where **h***_i_* 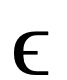 R*^d^* denotes the *i*-th word’s hidden state with a *d* dimension. A cell *c*_t_ is used to record the state of **h***_t_*; to control **h***_t_*, which is the data flow between the inputs and outputs, a forget gate *f_t_*, an output gate *o*_t_ and an input gate *i_t_* are employed. Where **W***_x_*, **U***_x_*, and *b_x_*(x 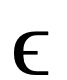 f, I, o, c) are model parameters, and *c*_0_ is a zero-filled vector:

*f_t_* = *σ*(*W_f_x_t_* + *U_f_h_t_*_–1_ + *b_f_*) **(1)**

*i_t_* = *σ*(*W_i_x_t_* + *U_i_h_t_*_–1_ + *b_i_*) **(2)**

*o_t_* = *σ*(*W_o_x_t_* + *U_o_h_t_*_–1_ + *b_o_*) **(3)**

*c_t_* = tanh(*W_c_x_t_* + *U_c_h_t_*_–1_ + *b_c_*) **(4)**

*h_t_* = *o_t_* • tanh(*c_t_*) **(5)**

It differs from the LSTM in that it uses a new function to replace the update function of the cell state *c_t_*. Specific ordering of internal neurons by replacing the update function, allowing the syntactic structure to be integrated into the LSTM. The update rules are as follows.



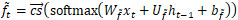

**(6)**




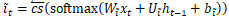

**(7)**




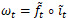

**(8)**


We used softmax to predict the layer order of neurons and then calculate the cumulative sum by cs. Finally, *f᷉_t_* and *i᷉_t_* contains the layer order information of *c_t_*_–1_ and *c_t_*, respectively, and the intersection of the two is *ω_t_*. The cumulative sum equation is as follows.



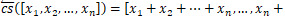





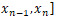

**(9)**




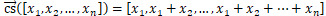

**(10)**


Following the cumulative sum’s properties, the master forget gate *f᷉_t_* has values that change from 0 to 1, while the master input gate *i᷉_t_* has values that decrease monotonically from 1 to 0. The overlap of *f᷉_t_* and *i᷉_t_* is represented by the product of the two master gates *ω_t_*.

*C* = *ω_t_* • (*f_t_* • *c_t_*_–1_ + *i_t_* • *c_t_*) + (*f᷉_t_* – *ω_t_*) • *c_t_*_–1_ + (*i᷉_t_* – *ω_t_*) • *c*_t_
**(11)**

Finally, the cell state *C* is segmented by layer order information, and the fused syntactic structure is fused in the model.

### Feature Processor

#### Multi-Head Attention

By building an attention adjacency matrix ***S****^k^*, we converted the feature **h** to a fully connected weight graph. A set of key-value pairs and a query were used in the calculation. The obtained attention matrices represent the potential syntactic tree, which is computed from the function of the keyword **K** with the corresponding query **Q**. In this case, both **Q** and **K** are the same as **h**.



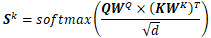

**(12)**


Where **W***^Q^* 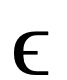 R*^d^*^×^*^d^* and **W***^K^* 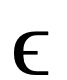 R*^d^*^×^*^d^* are parameters for projections, *d* denotes the vector dimension. **S***^k^* consists of 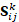. **h***_i_* and **h***_j_* represent the normalized weight scores of the *i*-th and the *j*-th token, respectively.

#### Matrix-Tree Pruning

We pruned the matrix-tree **S***^k^* to balance the syntactic features, output as matrix-tree A. It is achieved by multiplying a Gaussian kernel with an attention matrix. In the field of image processing, Gaussian kernel functions are commonly used to equalize images. In the model, we chose a 2-dimensional Gaussian kernel to balance the syntactic features. The following is the Gaussian kernel function.



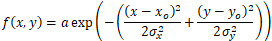

**(13)**


where *a* is the amplitude, *x_o_* and *y_o_* are the coordinates of the center point, and *σ_x_* and *σ_y_* are the variance. With the aforementioned 2-dimensional Gaussian kernel function, we could obtain the Gaussian kernel.

#### GCN

GCN is a neural network that can use information about the graph's structure. On the input of the GCN, we replaced the graph structure of the input with the syntactic tree matrix A generated above, and the feature vector is the output vector **h** of the Encoder. The layer-wise propagation rules of GCN are as follows:



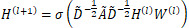

**(14)**


The adjacency matrix of an undirected graph **g** with extra self-connections is denoted by **Ã**, **Ã** = **A** + *I_N_. I_N_* is the identity matrix, *D᷉_ii_* = Σ*_i_* **Ã**_ij_. **W**^(^*^l^*^)^ is a trainable weight matrix. The activation function is denoted by *σ*(•). **H**^(^*^l^*^)^ 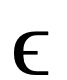 R*^N^*^×^*^D^* is the activation matrix in the *l*-th layer, **H**^(0)^ denotes the **h**.

### Classifier

To obtain final categorization representations, we combined sentence and entity representations and fed them into a feedforward neural network.

*H*_final_ = *FFNN*([*H_sent_* ; *H_s_* ; *H_o_*]) **(15)**

**H**_sent_, **H***_s_*, and **H***_o_* denote sentence, subject, and object representations, respectively. Finally, the logistic regression classifier performs predicted categorization of the outcome using **H**_final_ as a token.

## Results

### Results of the Cross-sentence Task

For the cross-sentence task, we used 3 types of models as baselines: (1) feature-based classifier [15] based on all entity pairs' shortest dependency pathways; (2) graph-structured LSTM methods, including bidirectional directed acyclic graph (DAG) LSTM (Bidir DAG LSTM) [5], Graph State LSTM (GS LSTM), and Graph LSTM [4]—these approaches extend LSTM to encode graphs generated from dependency edges created from input phrases; and (3) pruned GCNs [6] including attention-guided GCN (AGGCN) [14] and Lévy Flights GCN (LFGCN) [11]. These methods use GCNs to prune graphs with dependency edges. Additionally, we added the Bidirectional Encoder Representations from Transformers (BERT) pretraining model to complement the model with experiments. The results marked with an asterisk are based on a reimplementation of the original model.

In the multi-class relation extraction task (last 2 columns in Table 1), our SEGCN model outperforms all baselines with accuracies of 81.7 and 80.2 on all instances (Cross). In the ternary and binary relations, our SEGCN model outperforms the best performing graph-structured LSTM model (GS LSTM) by 10.0 and 8.5 points, respectively, our model outperforms the best performing model with LFGCN by 1.8 and 2.6 points when compared to the GCN models.

**Table 1:** Results of the cross-sentence task.

Model	Binary-class, accuracy				Multi-class, accuracy	
	Ternary		Binary		Ternary	Binary
	Single	Cross	Single	Cross	Cross	Cross
Feature-Based	74.7	77.7	73.9	75.2	—^a^	—
Graph LSTM^b^	77.9	80.7	75.6	76.7	—	—
DAG^c^ LSTM	77.9	80.7	74.3	76.5	—	—
GS LSTM^d^	80.3	83.2	83.5	83.6	71.7	71.7
GCN^e^ + Pruned	85.8	85.8	83.8	83.7	78.1	73.6
AGGCN^f^	87.1	87.0	85.2	85.6	80.2	77.4
LFGCN^g^	87.3	86.5	86.7	85.7	79.9	77.6
AGGCN + BERT^h^	87.2	87.1	86.1	84.9	80.5	78.1
LFGCN + BERT	87.3	86.5	86.5	86.7	80.3	78.0
SEGCN^i^	88.5	88.2	87.2	87.5	81.7	80.2
SEGCN + BERT	88.7	88.4	86.8	87.7	81.9	80.4

^a^Not determined.

^b^LSTM: long short-term memory.

^c^DAG: directed acyclic graph.

^d^GS LSTM: graph-structured long short-term memory.

^e^GCN: graph convolutional network.

^f^AGGCN: attention-guided graph convolutional network.

^g^LFGCN: Lévy Flights graph convolutional network.

^h^BERT: Bidirectional Encoder Representations from Transformers.

^i^SEGCN: syntactic edge-enhanced graph convolutional network.

In the binary-class relation extraction task, our SEGCN model also outperforms all baselines (first four columns in Table 1). The task was expanded to cross-sentence– (Cross) and sentence-level (Single) subtasks. In cross-sentence–level ternary and binary classification, our model received 88.2 and 87.5 points, respectively. Our model received 88.5 and 87.2 for sentence-level ternary and binary classifications, respectively.

These experiments show that our model achieves better results than previous models that discard shallow syntactic information, such as the previous GS LSTM and GCN models. We attribute the results of our models to the introduction of shallow syntactic information and the equalization process. Finally, for comparison with the latest methods, we attempted to introduce BERT pretraining. We found that the results of the task improved slightly after BERT pretraining. We believe that BERT also captured some shallow syntactic information during pretraining.

### Results of the Sentence-Level Task

The results of the sentence-level task using the CPR [11] and PGR [16] data sets are shown in Table . Our model has been compared to 2 types of models: (1) sequence-based models, including the randomly initialized Dilated and Depthwise separable convolutional neural network (Random-DDCNN) [9], which uses a parser that is a relational prediction model through random initialization and fine-tuning; attention-based multilayer gated recurrent unit [17], which overlays attentional mechanisms on top of the recursive gated units; Bran [18], which uses a bi-affine self-attention model to capture the sentence's interactions; and Bidirectional Encoder Representations from Transformers for Biomedical Text Mining [19], which is a pretrained language representation model for medical literature; and (2) dependency-based models, which are based on a single dependency tree, including the biological ontology–based long short-term memory network [20] and GCN. There are also dependency forest–based models, including the Edgewise–graph recurrent network (GRN) [8], which prunes scores greater than a threshold; kBest-GRN [8], which involves merging of k-best trees for prediction; ForestFT-DDCNN [9], which constructs a learnable dependency analyzer; and AGGCN and LFGCN [11], which relate multiheaded attention to dependency features.

**Table 2:** Results of the sentence-level task.

Type and model		Multi-class (BioCreative ViCPR data set), F1 score	Binary-class (Phenotype-Gene Relationship data set), F1 score
**Sequence-based model**			
	Random-DDCNN^a^	45.4	—^b^
	Att-GRU^c^	49.5	—
	Bran	50.8	—
	BioBERT^d^	—	67.2
**Dependency-based model**			
	BO-LSTM^e^	—	52.3
	GCN^f^	52.2	81.3
	Edgewise-GRN^g^	53.4	83.6
	kBest-GRN	52.4	85.7
	ForestFT-DDCNN	55.7	89.3
AGGCN^h^		56.7	88.5
LFGCN^i^		64.0	89.6
LFGCN+BERT		64.2	89.8
**Our models**			
	SEGCN^j^	65.4	91.3
	SEGCN+BERT	65.6	91.5

^a^DDCNN: Dilated and Depthwise separable convolutional neural network.

^b^Not determined.

^c^Att-GRU: attention-based multilayer gated recurrent unit.

^d^BioBERT: Bidirectional Encoder Representations from Transformers for Biomedical Text Mining.

^e^BO-LSTM: biological ontology–based long short-term memory.

^f^GCN: graph convolutional network.

^g^GRN: graph recurrent network.

^h^AGGCN: attention-guided graph convolutional network.

^i^LFGCN: Lévy Flights graph convolutional network.

^j^SEGCN: syntactic enhancement graph convolutional network.

As shown in the results of the sentence-level task in Table 2, our model achieved the best performance on both the multiclass data set CPR and the dichotomous data set PGR, with F1 scores of 65.4 and 91.3. Specifically, our model outperformed the previous state-of-the-art dependency-based model (LFGCN) by 1.2 and 1.5 points on the CPR and PGR data sets, respectively. We found that the model's improvement was smaller than that on the cross-sentence level task. We argue that shallow syntactic information has a smaller impact on short sentence lengths in sentence-level tasks, and it is better suited to long sentence lengths in cross-sentence tasks.

## Discussion

### Ablation Study

We validated the different modules of our model on the PGR data set, including BERT pretraining, the matrix-tree pruning layer, and the feature capture layer. Table 3 shows these results. We can see that model effectiveness decreases after removing any of the modules. All three modules can aid in the model's learning of a more accurate feature representation. The feature capture layer and the matrix-tree pruning layer improved by 2.4 and 2.5 points, respectively, indicating that the shallow syntactic information and equalization process resulted in a model boost. In contrast, the popular BERT pretraining approach was not suitable for the model.

**Table 3:** An ablation study using the Phenotype-Gene Relationship data set.

Model	F1 score
SEGCN^a^ (All)	91.5
SEGCN (- BERT Pretraining)	91.3
SEGCN (- Matrix-tree pruning)	90.0
SEGCN (- Feature capture)	89.1
Baseline (- All)	88.5

^a^SEGCN: syntactic enhancement graph convolutional network.

The ablation experiments show that shallow syntactic information and equalization processing methods can improve model performance significantly. We believe that these two methods function by processing the interaction information in the sentences. The shallow syntactic information complements the nonlocal interaction of the sentence, and the equalization process balances the local and nonlocal interactions of the sentence.

### Performance Against Sentence Length

**Figure 3:**
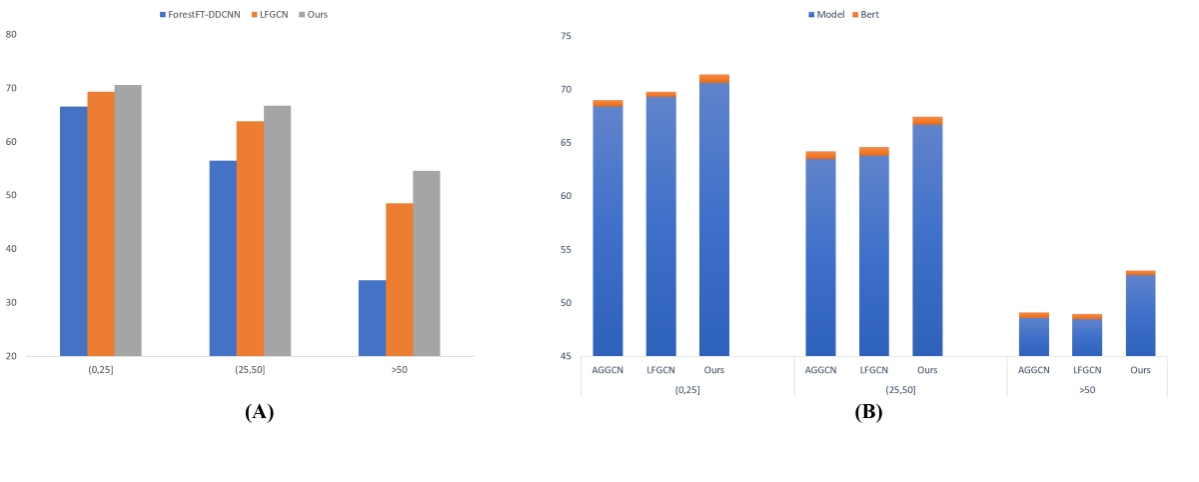
Performance against sentence length and Bidirectional Encoder Representations from Transformers (BERT) pretraining. (A) F1 scores at different sentence lengths. Results of the ForestFT– Dilated and Depthwise separable convolutional neural network are based on Jin et al [10]. (B) F1 scores against sentence length after BERT pretraining. AGGCN: attention-guided graph convolutional network; LFGCN: Lévy Flights graph convolutional network.

We examined the effect of introducing shallow syntactic information on different sentence lengths through comparative experiments. Figure 3A shows the F1 scores of the 3 models at different sentence lengths. There are 3 categories based on sentence length ((0,25), [25,50),>50). In general, our SEGCN outperformed ForestFT-DDCNN and LFGCN in all 3 length categories. Furthermore, the performance gap widened as the instance length increased. These results suggest that adding shallow syntactic information, particularly in long sentences, improves our model significantly. We attribute this to the fact that our model complements the nonlocal interactions of the sentences with the introduction of shallow syntactic information. Because they rely more on nonlocal interactions, longer sentences received higher F1 scores.

### Performance Against BERT Pretraining

To show the superiority of syntactic enhancement of our models, we compared the models with the addition of pretraining. After BERT pretraining, the F1 scores of the 3 models are shown in Figure 3B for different sentence lengths. There are 3 categories based on sentence length ((0,25], [25,50),>50). Overall, BERT pretraining showed small improvements for models of different sentence lengths. It supports our hypothesis that the neural network models acquire insufficient syntactic features. Furthermore, we found that our SEGCN without BERT still functioned better than the other models with BERT. These results indicate that our model outperforms BERT in using syntactical features.

### Case Study

**Figure 4:**
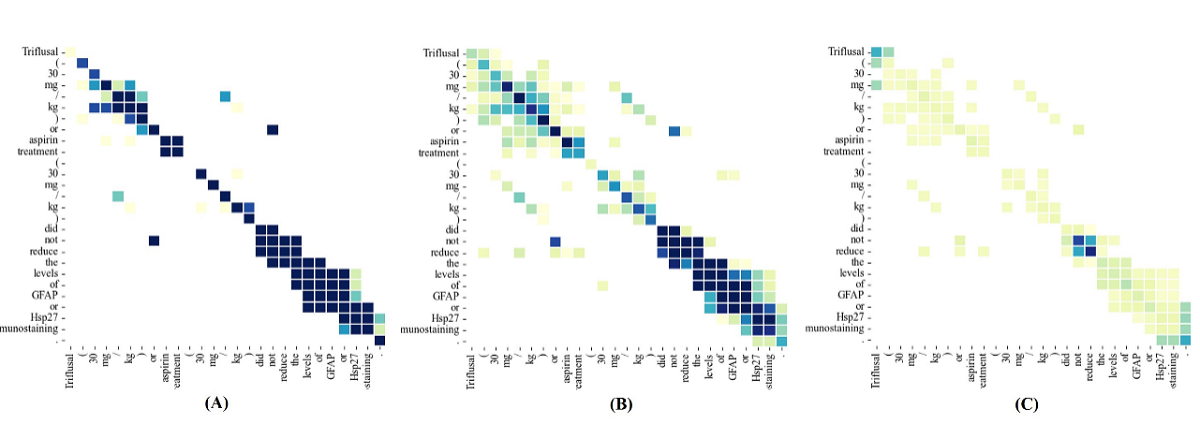
The heat maps of an example sentence in the syntactic enhancement graph convolutional network model.

To demonstrate the impact of our approach on sentence interaction, we compared the features obtained from different model layers. Figure 4 shows the attention weights of the example sentences at the different layers of the model. We decided to use a heat map to represent the attention weights. The color of each point represents the weight of the interactive information. The darker the color, the greater the weighting. For more intuition, we have omitted the points with smaller weights. In addition, the output of the multi-headed attention layer before and after incorporation into the shallow syntactic information is represented by matrices A and B, respectively. Matrix C represents the output of the equalization processing matrix B.

As shown in Figure 4, the weight distribution in matrix **A** is more concentrated in the diagonal distribution. In contrast, matrix B and matrix C have significantly more nondiagonal weight distributions than matrix A. This supports our view that the model incorporating shallow syntactic information gradually focuses on nonlocal interactions in the sentence. Furthermore, by comparing matrices B and C, we see that equalized matrix C pays more even-handed attention to the model's weights (the more similar the color, the closer the weights). We believe that the model's performance is improved by balancing the attention to local and nonlocal interactions. These results further demonstrate how our model makes use of syntactic information for syntactic enhancement.

### Conclusions

This study is the first to propose incorporating shallow syntactic information for syntactic enhancement in medical relation extraction. In addition, we devised a new pruning method to equalize the syntactic interactions in the model. The results for the 3 medical data sets show that our method can improve and equalize syntactic interactions, significantly outperforming previous models. The ablation experiments demonstrate the effectiveness of our two proposed methods. In future, we intend to continue our research on the connection between shallow syntactic information and sentence interactions.

## Abbreviations

AGGCN: attention-guided graph convolutional network
BERT: Bidirectional Encoder Representations from Transformers
DAG: directed acyclic graph
DDCNN: Dilated and Depthwise separable convolutional neural network
GCN: graph convolutional network
GRN: graph recurrent network
LFGCN: Lévy Flights graph convolutional network
LSTM: long short-term memory
ON-LSTM: ordered neuron–long short-term memory
PGR: Phenotype-Gene Relationship
Random-DDCNN: randomly initialized Dilated and Depthwise separable convolutional neural network
SEGCN: syntactic enhancement graph convolutional network
